# Supplementary material for: The Impact of Mindfulness Interventions upon Visual Attention and Attentional Bias Towards Food Cues: A Systematic Review
Source: Nutrients. 2025 Dec 12;17(24):3885. doi: 10.3390/nu17243885 (PMC12735511; doi:10.3390/nu17243885)
Supplement: Supplementary file 1 [file nutrients-17-03885-s001.zip › nutrients-3989841-supplementary.pdf]

Supplementary Materials

Table S1. Full-text database search strategy.

Database	String Search (keywords + Boolean Operators)	Filter
Web of Science	("mindful* eating" OR "mindfulness based eating awareness training" OR "MB-EAT" OR mindfulness OR “mindfulness intervention”) AND ("intervention" OR "practi?e" OR "approach" OR "program" OR "principle*" OR "behavi*r" OR "experiment*" OR "random* control* trial" OR "RCT") AND ("visual attention" OR "attentional bias" OR "eye tracking" OR "visual focus" OR "visual awareness" OR "visual concentration" OR "visual alertness" OR “visual processing” OR “visual bias” OR “cognitive bias” OR “eye movement” OR "dot probe task" OR "visual search task" OR "stroop task" OR "fixation duration" OR "fixation count" OR “gaze” OR “gaze pattern”) AND (food OR "food cue*" OR "food stimuli" OR "pictorial food cue*" OR "imaginal food" OR "physical food" OR "real food" OR "palatable food" OR "high* calori* food" OR "unhealthy food" OR "calori* dens* food" OR "high energy dens* food" OR "low calori* food" OR "healthy food" OR "low energy dens* food").	None
PubMed	(mindful* eating[Title/Abstract] OR "mindfulness-based eating awareness training"[Title/Abstract] OR “MBEAT”[Title/Abstract]) AND ("visual attention"[Title/Abstract] OR "attentional bias"[Title/Abstract] OR "attention bias"[Title/Abstract] OR "eye tracking"[Title/Abstract] OR "dot-probe"[Title/Abstract] OR "visual probe"[Title/Abstract] OR "dot probe task"[Title/Abstract] OR "visual search task"[Title/Abstract] OR "stroop task"[Title/Abstract] OR "fixation duration"[Title/Abstract] OR "fixation count"[Title/Abstract]) AND ("food cue"[Title/Abstract] OR "food image"[Title/Abstract] OR food stimul*[Title/Abstract] OR “food preference”[Title/Abstract]) OR "palatable food"[Title/Abstract]).	Randomised control trial, clinical trial, English
Scopus	TITLE-ABS-KEY ("mindful eating" OR "mindfulness-based eating intervention" OR "mindfulness-based eating awareness training" OR "MBEAT" OR "MB-EAT" OR mindfulness OR “mindfulness intervention”) AND TITLE-ABS-KEY ("visual attention" OR "attentional bias" OR “visual processing” OR “visual bias” OR “cognitive bias” OR	None

	“eye movement” OR "eye tracking" OR "dot-probe" OR "visual probe" OR "visual search task" OR "stroop task" OR "fixation duration" OR "fixation count" OR “gaze pattern”) AND TITLE-ABS-KEY ("food cue*" OR "food image*" OR "food stimulus*" OR "eating stimulus*" OR "food preference*" OR "palatable food*") AND NOT TITLE-ABS-KEY ("systematic review" OR"review article" OR review* OR "observational study" OR "cross sectional" OR "longitudinal").	
Springer Nature	("Mindful eating" OR "mindful* eating intervention" OR "mindfulness-based eating awareness training" OR “MB-EAT” OR mindfulness OR “mindfulness intervention”) AND ("intervention" OR "practi?e" OR "approach" OR "program" OR "principle*" OR "behavi*r" OR "experiment*" OR "random* control* trial" OR "RCT") AND (“attentional bias” OR "visual attention" OR “visual processing” OR “visual bias” OR “cognitive bias” OR “eye movement” "eye tracking?" OR "visual focus" OR "visual awareness" OR "visual concentration" OR "dot probe task" OR "visual search task" OR "stroop task" OR "fixation duration" OR "fixation count" OR “gaze pattern”) AND (food OR "food cues" OR "food stimuli" OR "food image*" OR "food task" OR "food image task" OR “palatable food” OR “unhealthy food” OR “healthy food”) NOT (“cross-sectional” OR “correlational” OR “longitudinal” OR “systematic review” OR “narrative review” OR “scoping review” OR “observational studies”).	None
MEDLINE	("mindful* eating" OR "mindfulness based eating awareness training" OR "MB-EAT" OR mindfulness OR “mindfulness intervention”) AND (“intervention*" OR "practi?e" OR "approach*" OR "program*" OR "principle*" OR "behavi*r" OR "experiment*" OR "random* control* trial*" OR "RCT") AND ("attention" OR “eye movements" OR "attention Bias") OR (“visual attention" OR "attentional bias" OR "eye tracking" OR visual bias” OR “cognitive bias” OR “eye movement” OR “visual focus" OR "visual awareness" OR "visual concentration" OR "visual alertness" OR "dot probe task" OR "visual search task" OR "stroop task" OR "fixation duration" OR "fixation count" OR “gaze pattern) AND ("food"OR "food preferences" OR "food habits"OR "feeding behavior") OR (food OR "food cue*" OR "food stimuli" OR "pictorial food cue*" OR "imaginal food" OR "physical food" OR "real food" OR "palatable food" OR "high* calori* food" OR "unhealthy food" OR "calori* dens* food" OR "high energy dens* food" OR "low calori* food" OR "healthy	None

	food" OR "low energy dens* food") NOT ("systematic review" OR"review article" OR review* OR "observational study" OR "cross sectional" OR "longitudinal").	
Embase	(mindful* eat* or "mindfulness based eating awareness training" or MB-EAT or mindfulness or "mindfulness intervention*" or "mindfulness training" or "mindfulness based intervention*").ti,ab. AND (intervention* or practi?e or approach* or program* or principle* or behav* or experiment* or random* control* trial* or RCT).ti,ab. AND ("visual attention" or "attentional bias" or attention* or "cognitive bias" or "information processing" or "attention control" or "selective attention" or "eye tracking" or "eye movement*" or "eye gaze" or "gaze pattern*" or "dot probe" or "visual search" or "stroop" or "fixation duration" or "fixation count").ti,ab. AND (food or eat* or "food cue*" or "food stimuli" or "food-related" or "eating-related" or "pictorial food cue*" or "imaginal food" or "palatable food" or "high* calori* food" or "unhealthy food" or "calori* dens* food" or "high energy dens* food" or "low calori* food" or "healthy food" or "low energy dens* food").ti,ab. NOT (cross-sectional or correlational or longitudinal or systematic review or narrative review or scoping review or observational stud*).ti,ab	None
CINAHL	("mindful* eating" OR "mindfulness based eating awareness training" OR "MB-EAT" OR mindfulness OR "mindfulness intervention") AND (intervention OR practi?e OR approach* OR program* OR principle* OR behavio?r OR experiment* OR "random* control* trial" OR RCT) AND ("visual attention" OR "attentional bias" OR "eye tracking" OR "eye movement*" OR "visual focus" OR "visual awareness" OR "visual concentration" OR "visual alertness" OR "dot probe task" OR "visual search task" OR "stroop task" OR "fixation duration" OR "fixation count" OR "gaze pattern*") AND (food OR "food cue*" OR "food stimuli" OR "pictorial food cue*" OR "imaginal food" OR "physical food" OR "real food" OR "palatable food" OR "high* calori* food" OR "unhealthy food" OR "calori* dens* food" OR "high energy dens* food" OR "low calori* food" OR "healthy food" OR "low energy dens* food") NOT ("cross-sectional" OR "correlational" OR "longitudinal" OR "systematic review" OR "narrative review" OR "scoping review" OR "observational stud*").	Peer reviewed

Note. Database searches conducted on September 10, 2025.

Table S2: Key findings for the six studies analysed.

Author, year, Country	Participant Population (N, age, % sex, BMI	Study Design, Experimental Conditions	Mindfulness Intervention	Outcome measure (primary and secondary)	Operational measures of visual attention or attentional bias	Key findings	Additional notes
Alamout et al.<br><br>(2020)<br><br>Iran	N = 45, not reported, 100% female<br><br>BMI = 27.28 ± 1.35 (at baseline)<br><br>Diet + MBCT group: 27.80 ± 1.62<br><br>Diet-only group: 26.96 ± 1.37<br><br>Control group: 27.07 ± 1.07	Between-subjects design with pretest-post-test-follow-up, randomised controlled with 3 groups (Diet + MBCT, Diet only, Control)<br><br>Diet + MBCT group (N = 15): Received an energy-restricted diet (≈ 800 kcal below daily intake) + 8 sessions of mindfulness-based cognitive therapy (MBCT)<br><br>Diet-only group (N = 15): received only diet therapy<br><br>Control group (N = 15): no intervention	Mindfulness-based Cognitive Therapy (MBCT)	Attentional bias to food cues (secondary)	Dot-probe task<br><br>Metrics: RT differences (ms)	MBCT along with the conventional diet therapy was more effective in decrease in attentional bias towards food cues than the diet therapy alone.<br><br>Diet + MBCT: Pretest: 3.76 ± 0.76 (ms). After 8 weeks: 2.08 ± 0.67 (ms). After 12 weeks: 1.98 ± 0.90 (ms).<br><br>Diet-only: Pretest: 3.72 ± 0.87 (ms). After 8 weeks: 3.97 ± 0.74 (ms). After 12 weeks: 3.74 ± 0.73 (ms).<br><br>Control: Pretest: 3.94 ± 0.65 (ms). After 8 weeks: 3.83 ± 0.47 (ms). After 12 weeks: 4.06 ± 0.56 (ms)	Age reported as inclusion criteria

Baquedano et al. (2017)<br><br>Chile	N = 50, 54% female. Age 23.9 ± 3.5, range 18-35	within-subjects design with 2 conditions<br><br>Immersed condition (encouraged to get immersed in food cues)<br><br>Mindful attention condition (encouraged to observe food cues with mindfulness)	Brief mindfulness attention induction task	Food approach bias (primary)<br><br>Salivary alpha-amylase (secondary)<br><br>Food Cravings Questionnaire (FCQ-S)<br><br>Five Facet Mindfulness Questionnaire (FFMQ; decentering subscale)<br><br>Cognitive Fusion Questionnaire (CFQ)	Approach avoidance task (AAT)<br><br>Metrics: RT differences (ms)	The mindful attention (MA) condition showed a significant reduction in food approach bias (FAB) to attractive and neutral food items compared to the immersed condition, $t(48) = 3.1$ $p < 0.01$	BMI not reported

Hussain et al.<br>(2022)<br>England<br>Study 1	N = 20, 75% female. Age 21.85 ± 3.18<br>BMI = 22.8 ± 5.14	Between-subjects experiment, randomised control trial with 2 groups<br>Mindfulness condition (N = 10, 70% female), participants listened to a 10-minute mindfulness meditation audio recording,<br>Control condition (N = 10, 80% female)	Mindfulness Meditation (MM)	Primary:<br>State Mindfulness (SMS-21)<br>Visual Attention/Attentional Bias (gaze duration and gaze directional bias)<br>Secondary:<br>Demographics (gender, age, height, weight ethnicity)<br>Hunger<br>BMI<br>Dietary Fat and Free Sugary Intake (DFS-26)	Screen-based eye-tracking (Tobii Pro X3-120)<br>Metrics: gaze duration, average fixation duration (ms), total fixation duration (ms).	MM increased state mindfulness and showed greater maintained attention toward LED food images vs HED food images compared to control.<br>Average fixation duration: $t(18) = 2.47$, $p = 0.02$<br>Total fixation duration: $t(18) = 4.47$, $p < 0.001$
Hussain et al.<br>(2022)<br>England<br>Study 2	N = 44, 86.36% female. Age 23.61 ± 6.87<br>BMI = 24.44 ± 4.67	Between-subjects experiment, randomised control trial with 2 groups<br>Mindful eating condition (N = 22, 95% female)<br>Control condition (N = 22, 77.27% female)	Mindful Construal Diary (MCD-R)	Primary:<br>State Mindfulness (SMS-21)<br>Visual Attention/Attentional Bias (gaze duration and gaze directional bias)	Screen-based eye-tracking (Tobii Pro X3-120)<br>Metrics: gaze directional bias, gaze duration bias, average fixation duration (ms), total	The MCD mindful-eating induction increased state mindfulness but did not change maintained attentional bias to food relative to control, favouring HED food items across

				Secondary:<br><br>Demographics (gender, age, height, weight ethnicity)<br><br>Hunger<br><br>BMI<br><br>Dietary Fat and Free Sugary Intake (DFS-26)	fixation duration (ms).	participants regardless of condition.<br><br>Participants displayed greater attentional bias towards HED food images compared to LED food images.<br><br>Gaze directional bias: $F(1, 42) = 4.83$, $p = 0.03$<br><br>Gaze duration bias: $F(1,42) = 5.54$, $p = 0.02$	
Mercado et al.<br><br>(2023)<br><br>England	N = 45, 78% female. Age 31.6 years.<br><br>BMI = 34.4 ± 6.57	between-subjects randomised controlled trial (8 weeks) with 3 groups<br><br>Mindfulness training condition (mindful eating)<br><br>Attention bias modification training condition (ABMT)<br><br>Waitlist control	Mindfulness training (eight weekly group sessions featuring brief meditations on mindful eating and cravings)	Primary: (feasibility**)**: Recruitment, retention, adherence.<br><br>Secondary: BMI, % body fat, EDE-Q, Power of Food Scale (PFS), Bogus taste test (food grams consumed)<br><br>Mood (DASS-21, STAI-S)	Dot-probe task + eye-tracking (Tobii Pro Fusion remote eye-tracking)<br><br>Metrics: RT differences (ms) + Initial fixation duration bias (ms), fixation duration (ms), saccadic latency (ms).	Only ABMT Significantly reduced attentional bias toward HED foods<br><br>Initial fixation duration: $t(12) = 2.981$, $p < 0.01$<br><br>Gaze duration bias: $t(12) = 3.378$, $p < 0.01$<br><br>MT Increased ME and decreased hedonic hunger, emotional eating, and disinhibition.	Age M, SD for total participants not reported. Participant BMI ranged from overweight to class III obesity. 47% had BED symptoms; 29% had psychiatric comorbidity

				Mindfulness (MAAS, MEQ)<br><br>Attention bias (Dot-probe with eye-tracking towards high- vs low-calorie food cues)			
Thomas et al. (2019)<br><br>United States	N = 51, 100% female. Age 57.92 ± 10.04,<br><br>BMI = 34.69 ± 7.39	Mixed-subjects, stage I pilot randomised controlled trial with 2 groups<br><br>Personalised Optimism with Exercise Recovery (POWER) Condition (10-week exercise + nutrition counselling program)<br><br>Mindfulness-Oriented Recovery Enhancement (MORE) + POWER Condition (10-week 1.5-hour sessions)	MORE (integrative treatment combining formal mindfulness meditation, cognitive reappraisal training, and savouring practices (aimed to increase interoceptive awareness, reappraise emotions, and amplify natural reward)	Interoceptive awareness (MAIA), maladaptive eating behaviours (DEBQ: restrained, emotional, external eating), savouring (Ways of Savouring momentary items), natural reward responsiveness (zygomatic EMG), food attentional bias (dot-probe), body composition/weight (weight, waist circumference, waist-to-hip ratio)	Dot-probe task<br><br>Metrics: mean RT differences (ms) towards food vs neutral image pairs (64 trial/ block).	MORE + POWER decreased automatic FAB (50ms cues) towards food images compared to POWER, $F(1, 28) = 12.21$, $p = .002$	88% of participants had breast cancer history**.**

Note. descriptive statistics are M and SD unless otherwise state

Table S3: Critical appraisal for the six included studies

			Item											
Author	Appraisal date	Design	1	2	3	4a, 4b, 4c	5	6	7	8	9	10	11	Appraisal
Alamout et al., 2020	11/10/2025	RCT	Y	Y	Y	U, U, U	Y	N	Y	Y	Y	Y	U	Moderate: 8/11
Baquedano et al., 2017	11/10/2025	RCT	Y	U	Y	N, N, U	N	N	Y	Y	U	U	U	High: 4/11
Hussain et al., 2022 Study 1	11/10/2025	RCT	Y	Y	Y	N, N, U	Y	Y	Y	Y	Y	Y	U	Low: 9/11
Hussain et al., 2022 Study 2	11/10/2025	RCT	Y	Y	Y	N, N, U	Y	Y	Y	Y	Y	Y	U	Low: 9/11
Mercado et al., 2023	11/10/25	RCT	Y	Y	Y	N, N, U	Y	Y	Y	Y	U	U	U	Moderate: 7/11
Thomas et al., 2019	11/10/2025	RCT	Y	Y	U	N, N, Y	Y	Y	U	Y	U	U	U	Moderate: 8/11

Note. Note. Y = Yes; N = No; U = Unclear/Can't tell. CASP (2024). Overall appraisal categories were derived using thresholds: Low (≥9/11), Moderate (6–8.5/11), and High (≤5.5/11).

Table S4: Critical appraisal summary for the six included studies

	Appraisal Summary		
Author	Positive Methodology	Negative Methodology	Unknowns
Alamout et al., 2020	• Random allocation to groups<br>• Mindfulness intervention (MBCT) sessions defined<br>• Outcome measures recorded at consistent time points<br>• Repeated measures with follow-up<br>• Appropriate statistical tests used (e.g., ANOVA) and reported	• Blinding for participants, investigators, and analysts not reported<br>• Small sample size (N = 45)<br>• Use of convenience sampling	• Blinding<br>• Details on randomisation
Baquedano et al., 2017	• Randomised order of within-subjects manipulations<br>• Use of validated stimulus set<br>• Physiological (e.g., DBP, SBP, salivary amylase) and behavioural (e.g., AAT) measures<br>• Appropriate statistical tests used (e.g., ANCOVA)	• No apparent participant randomisation to intervention vs control group<br>• Unequal distribution of males and females between groups<br>• No apparent blinding for participants<br>• Small sample size (N = 50	

Hussain et al., 2022<br>Study 1	• Use of validated questionnaire and stimulus sets<br>• Objective eye-tracking technology, calibration and usage failures<br>• Mindfulness interventions (MM) well defined<br>• Appropriate eye-tracking metrics, statistics, statistical tests used (e.g., ANOVA, ANCOVA)	• No apparent participant or investigator blinding to intervention/control group<br>• Unequal distribution of males and females across groups (<br>• Brief-single session mindfulness interventions<br>• Small sample sizes (N = 20)	Follow-up or long-term effects?
Hussain et al., 2022<br>Study 2	• Use of validated questionnaire and stimulus sets<br>• Objective eye-tracking technology, calibration and usage failures<br>• Mindfulness interventions (MCD) well defined<br>• Appropriate eye-tracking metrics, statistics, statistical tests used (e.g., ANOVA, ANCOVA)	• No apparent participant or investigator blinding to intervention/control group<br>• Unequal distribution of males and females across groups<br>• Brief-single session mindfulness interventions<br>• Small sample sizes (N = 44)	Follow-up or long-term effects?
Mercado et al., 2023	• Random allocation<br>• High session completion and participant completion rates	• Small sample size (N = 45)<br>• Lack of participant and investigator blinding	• Influence of response bias (e.g., potential for participant social desirability bias during questionnaires/ bogus taste-test

	• Use of objective eye-tracking technologies, appropriate eye-tracking metrics/ indices, use of validated questionnaires<br>• Use of appropriate statistical tests (e.g., ANOVA)	• Control group not comparable at follow-up<br>• Waitlist not assessed at follow-up<br>• No reporting of costs or resource data to assess value of feasibility	• Eye-tracking not measured at follow-up (do attentional biases persist commencing completion of intervention?)
Thomas et al., 2019	• Randomised allocation to condition/ control via computer software enabling blinding/ concealment from investigator/ testing staff<br>• Objective behavioural (dot-probe-attentional bias) outcome measures<br>• Appropriate use of attentional bias (RT) metrics/ indices<br>• Use of matched, active comparator (POWER + MORE/ POWER)	• Small sample size (N = 51)<br>• Notable participant attrition<br>• Lack of participant blinding<br>• CI% unreported<br>• Limited sample diversity	• Magnitude of whether attentional/ self-regulatory mechanistic changes sustained clinical outcomes in participant sample<br>• Influence of clinical group characteristics (e.g., cancer survivorship) upon responsivity to mindfulness intervention (MORE)
